# The reliability and validity of a visual analog scale for rating the single leg squat test

**DOI:** 10.7717/peerj.21600

**Published:** 2026-07-28

**Authors:** Yongni Zhang, Yifan Liu, Xinwei Huang, Christopher R. Carcia, RobRoy L. Martin, Zhicheng Pan

**Affiliations:** 1 Duquesne-China Health Institute, Rangos School of Health Sciences, Duquesne University, Pittsburgh, PA, United States of America; 2 Department of Rehabilitation Therapy, Shanghai Yangzhi Rehabilitation Hospital (Shanghai Sunshine Rehabilitation Center), School of Medicine, Tongji University, Shanghai, China; 3 Department of Kinesiology, Colorado Mesa University, Colorado, United States of America; 4 Physical Therapy, Duquesne University, Pittsburgh, PA, United States of America; 5 UPMC Center for Sports Medicine, Pittsburgh, PA, United States of America; 6 Bone and Joint Rehabilitation Center, Shanghai Yangzhi Rehabilitation Hospital (Shanghai Sunshine Rehabilitation Center), School of Medicine, Tongji University, Shanghai, China

**Keywords:** Minimal detectable change, Minimal detectable difference, Visual assessment, Inter-rater reliability, Intra-rater reliability, Functional performance test

## Abstract

**Background:**

The single leg squat test (SLST) is commonly used for visual movement assessment in subjects with lower extremity injuries. While current methods utilize ordinal rating categorization, adopting a visual analog scale (VAS) may offer potential advantages. The study aims to determine the intra-rater, inter-rater reliability, and validity of a visual analog scale (VAS) for rating the single leg squat test (SLST) in subjects with lower extremity injuries.

**Methods:**

58 subjects with lower extremity injuries participated. Intra-rater reliability was assessed by a physical therapist at 1-week and 2-week intervals. Single session inter-rater reliability was assessed by two physical therapists. A 10 cm VAS was used to measure trunk lateral flexion, hip adduction, and dynamic knee valgus during the SLST. Intra-class correlation coefficients (ICC) assessed intra-rater and inter-rater reliability. Validity was assessed by comparing VAS scores to 2-D kinematic analysis.

**Results:**

Intra-rater reliability was good at both 1 week (ICC = 0.81–0.92, MDC = 1.2–2.2 cm) and 2 weeks (ICC = 0.77–0.79, MDC = 2.7–3 cm). Inter-rater reliability was good to excellent (ICC = 0.78–0.93, MDD = 1–2.6 cm). The correlation between visual assessment and 2-D kinematic analysis was moderate to good (*r* = 0.66–0.78).

**Conclusion:**

Preliminary data indicate that VAS reliably and validly rates SLST in individuals with lower-extremity injuries, allowing calculation of minimal detectable change and minimal detectable difference to support clinical interpretation. The MDC and MDD values established for trunk lateral flexion, hip adduction, and dynamic knee valgus allows clinicians to objectively determine if scores between repeated measurement and two raters are different.

## Background

Assessing individuals for movement system dysfunction is important when evaluating musculoskeletal pathologies and screening for injury prevention (Carroll, Paulseth & Martin, 2022; Disantis & Martin, 2022a; Disantis & Martin, 2022b; Zarzycki et al., 2022). The single leg squat test (SLST) is commonly used for assessment in those with impairments of strength, balance, and/or neuromuscular control of the trunk and lower extremity (Hatton et al., 2014; Lewis et al., 2015; McGovern et al., 2018). Although there is evidence to support the use of the SLST, considerable variation exists in how the test is performed and interpreted (McGovern et al., 2018; Ressman, Grooten & Rasmussen Barr, 2019). The current SLST rating methods using primary ordinal scoring does not easily allow clinicians to interpret changes in SLST performance over time.

The SLST has gained widespread popularity in clinical practice as an easy to perform functional assessment, with biomechanical and neuromuscular similarities associated with more complex movements (McGovern et al., 2018; Nae et al., 2017; Ressman, Grooten & Rasmussen Barr, 2019). Assessments typically involve rating movement control of the trunk and lower extremity. Evidence supports the use of the SLST for those with hip arthritis (Lenzlinger-Asprion et al., 2017), non-arthritic intra-articular hip conditions (Martin et al., 2023; McGovern et al., 2019; McGovern et al., 2018), greater trochanteric pain syndrome (Ferrer-Peña et al., 2020), anterior cruciate ligament reconstruction (Hall et al., 2015; Madhavan & Shields, 2011), knee arthritis (Kaukinen et al., 2017; Mastrigt et al., 2017) and patellofemoral pain (Crossley et al., 2011; Hansen, Lundgaard-Nielsen & Henriksen, 2021; Herrington, 2014; Levinger, Gilleard & Sprogis, 2006; Nakagawa et al., 2012; Willson & Davis, 2008) as well as a screening tool in healthy individuals (Gianola et al., 2017; Harris-Hayes et al., 2014; Poulsen & James, 2011; Weeks, Carty & Horan, 2012; Whatman, Hing & Hume, 2012; Whatman, Hume & Hing, 2013). Additionally, the SLST performance has been linked to impairments of trunk and hip musculature (Boudreau et al., 2009; Claiborne et al., 2006; Hollman et al., 2014; Kivlan & Martin, 2012; McGovern et al., 2020; Nakagawa et al., 2012; Stickler, Finley & Gulgin, 2015). However, systematic reviews have not identified the best way to administer and rate an individual’s performance with the SLST (Nae et al., 2017; Ressman, Grooten & Rasmussen Barr, 2019). A meta-analysis by Ressman, Grooten & Rasmussen Barr (2019) identified more than 10 versions of the SLST, with variations in name, protocol, and rating assessment methods. SLST rating approaches range from a global assessment that evaluates trunk, pelvis, hip, knee, and ankle alignment together to separate assessments of each of these five components (Ressman, Grooten & Rasmussen Barr, 2019). Similarly, methods used to rate movement quality are varied with ordinal scales ranging from two to 10 categories (Ressman, Grooten & Rasmussen Barr, 2019; Zhang et al., 2025c). Despite this variation in how the SLST is administered, it does have evidence for intra- and inter-rater reliability with little difference in reliability based on the number of components and rating methods (Ressman, Grooten & Rasmussen Barr, 2019).

All of the methods used to visually rate the SLST have been described using ordinal scales to define an individual’s performance (Ressman, Grooten & Rasmussen Barr, 2019). A visual analog scale (VAS) with the use of continuous data has potential advantages over ordinal rating scales. Most notably continuous data allows for standard error of measure (SEM), minimal detectable difference (MDD), minimal detectable change (MDC), minimal clinically important difference (MCID) and substantial clinical benefit (SCB) values to be defined. These values are useful clinically when assessing for a patient’s change in status. The purposes of this study were to determine if the SLST could be rated using a VAS and if SEM, MDC and MDD values could be determined in subjects with lower extremity injuries. It was hypothesized that rating the SLST using the VAS would demonstrate good intra-rater reliability and inter-rater reliability and acceptable validity compared to 2-D video analysis.

## Materials & Methods

This cross-sectional study involved two steps. Step one involved assessing the intra-rater reliability and validity of visual rating for SLST. Intra-rater reliability was determined by comparing the independent rating of a physical therapist with 11 years of experience at 1-week and 2-week time intervals. The validity of visual rating was determined by comparing the therapist’s ratings with those obtained from 2-D video recordings at baseline, captured simultaneously during the therapist’s visual evaluation. Step two involved assessing the inter-rater reliability of visual rating for SLST assessment by comparing the simultaneous independent ratings of two experienced physical therapists (35 years and 11 years). The study was approved by Duquesne University Institutional Review Board (Protocol ID: 2022/02/14). Written informed consent was obtained from all subjects prior to their enrollment in the study.

### Participants

Based on a power analysis assuming an ICC of 0.75, two raters (*k* = 2), and the requirement that the two-sided 95% confidence interval’s lower bound ≥ 0.60, a one-way random-effects ICC(1,2) design required a sample size of 29 subjects (Shrout & Fleiss, 1979). A total of 58 subjects diagnosed with lower extremity injuries participated in this two-step study, with 29 participants enrolled in the intra-rater reliability and validity analyses (step one) and a separate group of 29 participants enrolled in the inter-rater reliability analysis (step two). After signing the consent form subjects provided their demographic and medical history information. Inclusion criteria were 18 to 45 years of age with a lower extremity injury (hip, knee, foot, or ankle) diagnosed by an athletic trainer, physician, or physical therapist within the last 6 months. Exclusion criteria included recent surgery with weight bearing precautions, neurological disorder, ambulation requiring an assistive device, or inability to perform the SLST.

### Data collection

Data collection procedures were performed as previously described by Zhang et al. (2025c). Subjects completed the Lower Extremity Functional Scale (LEFS) and University of California at Los Angeles (UCLA) Activity Scale before the SLST. The LEFS has appropriate psychometric properties for assessing lower extremity functional performance (Mehta et al., 2016; Zhang et al., 2025a; Zhang et al., 2025b; Zhang, Zang & Martin, 2025; Binkley et al., 1999). The UCLA Activity Scale has appropriate psychometric properties for assessing physical activity levels (Naal, Impellizzeri & Leunig, 2009; Rolfson et al., 2016; Terwee et al., 2011).

**Figure 1:**
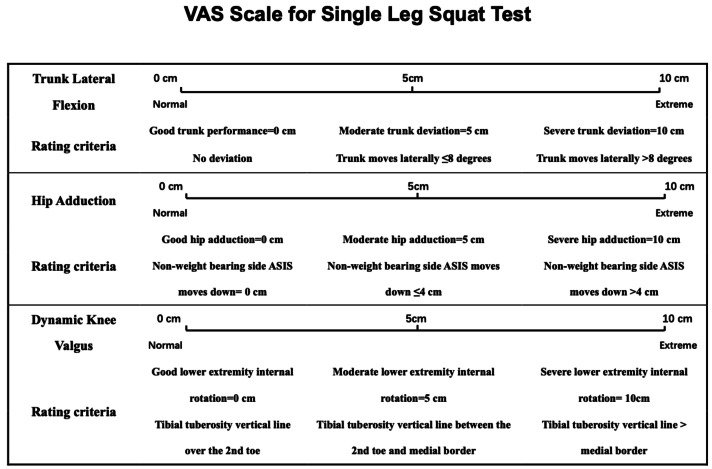
Visual analog scale scoring criteria for single leg squat test. VAS scale for single-leg squat assessing trunk, hip, and knee deviations from normal (0 cm) to extreme (10 cm), based on movement and alignment criteria.

A 15-minute practice rating session was performed by the raters to familiarize themselves with the rating criteria and SLST VAS (Fig. 1) prior to data collection. This session involved volunteer practice participants who were not included in the study sample and was used to standardize scoring procedures and interpretation of the rating criteria. The SLST VAS was scored, using three 10-centimeter VAS lines with 0 cm representing no deviation and 10 cm representing extreme deviation (*I.e.*, maximal deviation before falling), by physical therapists when assessing the components of trunk lateral flexion, hip adduction and dynamic knee valgus during the SLST (Fig. 1). The rating criteria for the SLST in this study were based on McGovern et al.’s (2018). literature review. For the intra-rater reliability step, a therapist rated subjects’ SLST performance using the VAS scale for deviation at baseline, 1 week, and 2 weeks after baseline. Baseline SLST performance was recorded with 2-D video while the therapist conducted the visual assessment. Video was captured with an iPad air 4 (apple, US) at 60 frames per second (Krause et al., 2015). The iPad was supported perpendicular to the ground, at a height of 80 cm and a distance of 140 cm from the subject. For inter-rater reliability, two physical therapists simultaneously and independently rated subjects’ SLST performance.

### SLST procedures

Both the intra-rater and inter-rater reliability steps followed the same SLST procedures. Subjects were instructed to wear shorts/tights and a T-shirt. Subjects received verbal instructions and demonstration on how perform the SLST, as described by McGovern et al. (2018). A visual description of the testing procedure is provided in the Appendix S1. In summary, subjects were instructed to stand barefoot with both legs shoulder-width apart, parallel to each other, and arms positioned at their side. They placed their injured foot on the long axis of the “T” shape with the second metatarsal aligned perpendicular to the stem but not touching the line. Subjects then transitioned to a single leg-stance on the injured leg, flexing the non-stance knee to 90° and vertically aligning the thigh with the stance leg. Subjects were instructed to maintain a straight trunk while squatting to a depth at which they could no longer see the line in front of their toes (around 60° of knee flexion), maintaining a balanced and controlled motion at an approximate rate of one squat per two seconds. During the squat, subjects were instructed to mentally count “1001, 1002” from the beginning to the end of each repetition to help pace the 2-second duration. Before testing, subjects were asked to practice the SLST three times successfully on the test leg.

After the three practice trials, subjects were asked to perform three consecutive SLSTs which were assessed by the raters. If a subject lost their balance, the trial was discarded, and the subject repeated the trial. Raters marked the three VAS corresponding to each of the three components (trunk lateral flexion, hip adduction, dynamic knee valgus) to define SLST performance. Ratings were based on the worst performance for each component across three repetitions of the SLST (Fig. 1) (Zhang et al., 2025c).

### Data processing

During the assessment, the therapists placed a mark on the VAS rating scale to indicate their evaluation. After the completion of all testing sessions, one researcher collected scales and measured the distance from the zero point to therapists’ mark using a tape measure to extract the visual assessment data. Two researchers independently extracted kinematic data from the 2-D video. The consistency between their extractions was assessed using a two-way random model ICC (2, 1), and the measurement error was quantified by SEM. All 2-D kinematic analysis of knee, hip and trunk in this study was performed using Image J software as previously described by Kingston et al. (2020). Figures illustrating the calculation of angles during 2-D video analysis is provided in the Appendix S1. The moments of maximum deviation were determined by having the rater review the video recordings of each squat trial in slow motion. The rater identified the frame in which the maximum deviation of the knee, hip, or trunk occurred during the movement. This frame, representing the point of greatest deviation, was captured as a still image and used for subsequent analysis. The knee Frontal Plane Projection Angle (FPPA) was used to assess the correlation with visual assessment on dynamic knee valgus. The angular differences between the hip FPPA and Lateral Trunk Motion at the start of the squat and at their respective points of maximum deviation during the squat were used to assess the correlation with visual assessments on hip adduction and trunk lateral flexion, respectively. Knee FPPA was calculated by drawing two lines: one from the anterior superior iliac spine (ASIS) to the center of the patella, and the other from the center of the patella to the center of the ankle mortise. The angle formed at the patella between these two lines was subtracted from 180 degrees to determine the knee FPPA. Hip FPPA was calculated by drawing two lines: one connecting bilateral ASIS and another from the center of the patella to one of the ASIS on the testing limb. The Lateral Trunk Motion was determined by measuring the angle between two lines: one drawn from the ASIS of the testing limb perpendicular to the ground, and the other from the ASIS of the testing limb to the midpoint between the two acromioclavicular joints.

### Data analysis

VAS SLST scores collected at one- and two-week intervals were compared to baseline measurements to calculate intra-rater reliability. Data from the two physical therapists were used to calculate inter-rater reliability. Intraclass correlation coefficients (ICCs) were calculated using a two-way random-effects model (ICC [2, 1]) for inter-rater reliability and a two-way mixed-effects model (ICC [3, 1]) for intra-rater reliability. ICC values were interpreted according to the scale described by Koo & Li (2016) (*i.e.,* poor reliability 0–0.5; moderate reliability 0.5–0.75; good reliability 0.75–0.9 and excellent reliability 0.9–1.00). The SEM values were calculated as follows: SEM = SD_diff_√(1 − ICC) with SD_diff_ representing the standard deviation of mean difference between two measurements (De Vet et al., 2006; Stratford & Riddle, 2012). The MDC and MDD values were calculated as follows: MDC = *z*-score (95% CI) × SEM × $\sqrt{2}$ and MDD = *z*-score (95% CI) × SEM × $\sqrt{2}$ (Haley & Fragala-Pinkham, 2006). The MDC values represent the magnitude of measurement error between repeated assessments, while MDD values represent the measurement error between two raters (Haley & Fragala-Pinkham, 2006; Martin & Irrgang, 2007).

For construct validity, baseline kinematic data from 2-D video analysis and baseline VAS SLST scores, assessed by the therapist who performed the intra-rater VAS evaluations in step one, were used. Pearson or Spearman correlation coefficients were selected based on the distributional characteristics of the data. Correlation values were interpreted according to the scale described by guidelines (*i.e.,* weak correlation 0–0.30; moderate correlation 0.30–0.49; and strong correlation above 0.5) (Brydges, 2019). All statistical calculations were completed using the SPSS Version 29 (IBM; Armonk, NY).

## Results

Subject demographic information is presented in Table 1. The results for intra-rater and inter-rater reliability, as well as MDC and MDD values for SLST using the VAS to assess trunk lateral flexion, hip adduction, and dynamic knee valgus, are presented in Table 2. At 1-week, intra-rater reliability was good (ICC = 0.81–0.92) with MDC values ranging between 1.2–2 cm for trunk lateral flexion, hip adduction, and dynamic knee valgus. At 2 weeks, intra-rater reliability was good (ICC = 0.77–0.79) with MDC values ranging between 2.7–3 cm for trunk lateral flexion, hip adduction, and dynamic knee valgus. Inter-rater reliability was also good to excellent (ICC = 0.78–0.93) with MDD values ranging between 1–2.6 cm for trunk lateral flexion, hip adduction, and dynamic knee valgus.

**Table 1:** Demographic information for subjects.

Variable	Intra-rater reliability	Inter-rater reliability
*N*	29	29
Age	25.4 years (SD = 5.4)	23.1 years (SD = 3.2)
Height	171 cm (SD = 8.8)	169.7 cm (SD = 7)
Body mass	70.8 kg (SD = 18.6)	66.9 kg (SD = 9.9)
Female	16 (55.2%)	18 (62%)
Male	13 (44.8%)	11 (38%)
LEFS	65.6 (4.4)	66.2 (3.6)
Right leg involved	13 (44.8%)	19 (65.6%)
Left leg involved	16 (55.2%)	10 (34.4%)
UCLA level		
4–6	10 (34.5%)	13 (44.8%)
8–10	19 (65.5%)	16 (55.2%)
Region of injury		
Intra-articular hip	1 (3.4%)	2 (6.8%)
Extra-articular hip	2 (6.8%)	2 (6.8%)
Intra-articular knee	4 (13.8%)	9 (30.9%)
Extra-articular knee	8 (27.6%)	8 (27.5%)
Foot and ankle	14 (48.2%)	8 (27.5%)

**Table 2:** Evidence of reliability for the visual analog scale single leg squat test ratings.

Components	Intra-rater reliability 1 week		Intra-rater reliability 2 week		Inter-rater reliability	
	ICC (95% CI)	MDC (cm)	ICC (95% CI)	MDC (cm)	ICC (95% CI)	MDD (cm)
Trunk lateral flexion	0.85 (0.71, 0.92)	2	0.79 (0.61, 0.9)	3	0.84 (0.69, 0.92)	1.2
Hip adduction	0.92 (0.83, 0.96)	1.2	0.77 (0.57, 0.89)	2.7	0.78 (0.58, 0.89)	2.6
Dynamic knee valgus	0.81 (0.64,0.9)	2.2	0.79 (0.6, 0.89)	3	0.93 (0.86, 0.96)	1

**Notes.**

ICC Intra-class correlation coefficients MDC Minimal Detectable Change MDD Minimal Detectable Difference

Shapiro–Wilk testing indicated that the VAS variables were not normally distributed (all *p* < .05), whereas the 2D measures demonstrated approximate normality (all *p* > .05). The validity between 2-D kinematic analysis and VAS ratings of SLST by the physical therapist was moderate to strong for trunk lateral flexion, hip adduction, and dynamic knee valgus. The *ρ* values were 0.77 (95% CI [0.57–0.89]) for trunk lateral flexion, 0.5 (95% CI [0.39–0.73]) for hip adduction, and 0.84 (95% CI [0.69–0.92]) for dynamic knee valgus. Scatter plots of correlation are presented in Appendix S1. The inter-rater reliability between the two researchers who independently extracted kinematic data from the 2-D video was excellent, with ICC values ranging from 0.95 to 0.97 and SEM ranging from 0.6 to 1.7 degrees for trunk lateral flexion, hip adduction, and dynamic knee valgus (Appendix S1).

## Discussion

This study provides preliminary evidence of intra-rater and inter-rater reliability, as well as validity, supporting the use of SLST with three VAS scales for trunk lateral flexion, hip adduction, and dynamic knee valgus in young, active individuals with a recent history of lower extremity injury. Furthermore, the MDC and MDD values established for the SLST VAS will allow clinicians to objectively determine whether SLST score differences exceed measurement error, either between repeated assessments at 1- and 2-week intervals or between two raters. The SLST VAS methods used in this study provide advantages to the previously used ordinal scales and may allow researchers to use continuous data from the SLST to define other clinically useful values such as MCID and SCB. However, these findings were derived from a small group of experienced raters and a relatively homogeneous population. Therefore, caution is warranted when generalizing the results beyond similar clinical settings.

The meta-analysis by Ressman, Grooten & Rasmussen Barr (2019) identified two studies that investigated the intra-rater reliability of real-time SLST visual assessment (Cornell & Ebersole, 2018; Ortqvist et al., 2011). One study, using a proprietary algorithm to generate a continuous score from categorical data (compensations/no compensations), reported a moderate intra-rater reliability in healthy subjects, with ICC of 0.55 for overall SLS performance over a one day interval (Cornell & Ebersole, 2018). Another study reported moderate intra-rater reliability in healthy subjects, with Kappa of 0.48 over 7 to 10 days, using categorical data to rate the knee component as medial, neutral, or lateral during the SLST (Ortqvist et al., 2011). The current study found good intra-rater reliability among young, active individuals with a recent history of lower extremity injury, with ICCs ranging from 0.77 to 0.92 over one and two week time interval. Using the three components of trunk lateral flexion, hip adduction, and dynamic knee valgus makes direct comparison with these two studies challenging. In the current study, intra-rater reliability was slightly lower at 2 weeks (ICC = 0.77–0.79) than at 1 week (ICC = 0.81–0.92). This difference may reflect true changes in participant performance over time rather than measurement error alone, particularly in individuals with recent lower extremity injuries. To our knowledge, this study is the first to define MDC values for the visual assessment of the SLST, providing clinicians and researchers with objective thresholds to determine whether observed changes exceed measurement error. Furthermore, the current study assessed intra-rater reliability at 1-week and 2-week intervals, offering a more clinically relevant timeframe for practice.

In addition to intra-rater reliability, the meta-analysis also identified six studies that analyzed real-time visual assessment of inter-rater reliability (Ressman, Grooten & Rasmussen Barr, 2019). These studies used ordinal scales with two, (Ageberg et al., 2010; McGovern et al., 2019; Ortqvist et al., 2011) three (Fridrich et al., 2017) and four (Frohm et al., 2012; Junge et al., 2012) categories. Three studies analyzed the total scores of ordinal categories and found ICC values ranging from 0.14 to 0.93 in healthy subjects and those with nonarthritic hip pain (Fridrich et al., 2017; Frohm et al., 2012; McGovern et al., 2019). The other three studies found Kappa values ranging from 0.54 to 0.92 in healthy subjects (Ageberg et al., 2010; Junge et al., 2012; Ortqvist et al., 2011). The current study found moderate to excellent inter-rater reliability with ICC values ranging from 0.78 to 0.93 for the three individual component scores in young, active individuals with a recent history of lower extremity injury. The two studies that had comparably high inter-rater reliability used either experienced clinicians or practice sessions prior to testing (Ageberg et al., 2010; McGovern et al., 2019). Similar to this current study, McGovern et al. (2019) included two experienced clinicians and found excellent inter-rater reliability (ICC = 0.93) for overall score using ordinal scales for the trunk, hip, and lower extremity in subjects with nonarthritic hip pain. Also similar to this current study, Ageberg et al. (2010) included rater practice sessions and found excellent inter-rater reliability (Kappa = 0.92) using a two categorical ordinal scale in healthy subjects. To our knowledge, this study is the first to define MDD values for visual assessment of the SLST, providing objective thresholds to determine whether differences in scores between raters exceed the measurement errors.

The high intra-rater and inter-rater reliability on SLST VAS in this current study may be attributed to the raters’ experience, practice sessions prior to testing, and the number of rating components. A previous study found that experienced physiotherapists achieved higher reliability and validity than students in assessing SLS performance from videos in healthy subjects (Weeks, Carty & Horan, 2012). Three studies reported the use of experienced clinicians to rate the SLST but found low inter-rater reliability. However, their practice sessions were either not detailed or not included (Fridrich et al., 2017; Frohm et al., 2012; Ortqvist et al., 2011). Another key difference between the current study and previous studies lies in the separate assessment of trunk, hip, and lower extremity deviations. In contrast, other studies primarily focused on overall performance or composite scores of two or more components (Cornell & Ebersole, 2018; Fridrich et al., 2017; Frohm et al., 2012; Junge et al., 2012). The rating method employed by this current study, which examined specific segmental deviations at the trunk, hip, and lower extremity, may potentially allow relationships to be made between abnormal biomechanical performance and neuromuscular function in these three distinct body regions. This may have potential benefit to direct interventions at a specific body region in an effort to improve a patient’s neuromuscular control.

In the current study, real-time visual assessment of SLST was compared to 2-D kinematic analysis to establish validity. Two raters were able to independently extract 2-D kinematic data with a measurement error of less than 2 degrees. The results showed good correlations between kinematic data and VAS SLST, with *ρ* values of 0.77, 0.5, and 0.84 for trunk lateral flexion, hip adduction, and dynamic knee valgus, respectively. The confidence intervals for the *ρ* values across all variables were wide ranging from 0.39 to 0.92, indicating that the interpretation of the method’s validity should be made with caution. Similar validity has been reported in previous studies (Ageberg et al., 2010; Whatman, Hume & Hing, 2013). Ageberg et al. (2010) found in healthy subjects who were rated to have knee valgus on real time visual assessment showed greater knee valgus on 2-D kinematic analysis. Similarly, Whatman, Hume & Hing (2013) using video analysis found those who performed the SLST with a patella media to 2nd toe had an increased peak in knee FPPA on 2-D kinematic analysis. Although the current study found strong Spearman correlations, the association for the hip adduction component was weaker than those for the trunk and knee components. This finding suggests that hip motion may be more difficult to visually detect during real-time assessment. The lower confidence bound (*ρ* = 0.39) for the hip adduction component suggests uncertainty in its validity estimate compared with the trunk and knee components.

In contrast to the previous studies that used ordinal rating scales, the VAS from this study allowed for the collection of continuous data and the calculation of MDC and MDD values, allowing therapists to judge whether observed change exceeds measurement error. Clinically, this approach enables real-time, equipment-free assessment of SLST performance with quantifiable thresholds for meaningful change. MDC values represent the magnitude of measurement error between repeated assessments (Haley & Fragala-Pinkham, 2006). According to the results of this study, if a clinician rated a patient as five cm on each of the three VAS scales, the scores would be considered improved at the 1-week retest if they are lower than three cm for trunk lateral flexion, 3.8 cm for hip adduction, and 2.2 cm for dynamic knee valgus on the 10 cm VAS. Similarly, improvement at the 2-week retest would be indicated by scores lower than two cm for trunk lateral flexion, 1.3 cm for hip adduction, and two cm for dynamic knee valgus. MDD values represent the smallest amount of difference that exceeds the measurement error between two raters (Martin & Irrgang, 2007). According to the results of this study, if a clinician rated a patient as five cm on each of the three VASs, a second examiner would be expected to rate the subject between 6.2 or 3.8 cm for trunk lateral flexion, 7.6 or 2.4 cm for hip adduction, and 6 or 4 cm for dynamic knee valgus.

## Limitations

There are limitations associated with methods of this study that could influence the generalizability of the results. First, intra-rater reliability was assessed by a single physical therapist, and inter-rater reliability by only two therapists. Although these raters were experienced and received prior training, the small number of raters limits the ability to generalize the findings to a broader clinical population. Second, experienced physical therapists performed practice sessions on the rating criteria of the SLST on several volunteers before the data collection session. Implementing this approach in a clinical setting might be challenging; however, it highlights the potential value of training sessions on improving the reliability in rating the SLST. Because both raters in the current study had substantial clinical experience, these findings may not generalize to novice clinicians or students, who may require additional training before achieving similar reliability. Third, this study focused on a young and active population with a history of various lower extremity injuries. However, the inclusion of those with a variety of lower extremity injuries may help to improve generalizability. Further studies can be done on larger populations with specific pathologies. As this was an initial attempt to develop a VAS rating system for the SLST, the primary aim was to establish preliminary feasibility rather than to perform large-scale validation. It is important to note that the SLST is a broadly applicable assessment tool that can be used across a wide range of populations, including both individuals with different musculoskeletal conditions and healthy individuals.

## Conclusion

This study presents a preliminary exploration of a novel method for rating the SLST using a VAS in young, active individuals with a recent history of lower extremity injury. The findings suggest that VAS-based scoring may yield good to excellent intra- and inter-rater reliability for trunk lateral flexion, hip adduction, and dynamic knee valgus when used by a small sample of experienced raters. The continuous data for rating the SLST allowed for establishing MDC and MDD values for trunk lateral flexion, hip adduction, and dynamic knee valgus. However, the limited number of raters restricts the generalizability of these findings, and further studies with larger and more diverse rater samples are needed to confirm these initial results.

##  Supplemental Information

10.7717/peerj.21600/supp-1 Appendix S1 Appendix

10.7717/peerj.21600/supp-2 Dataset S1 Dataset

## Additional Information and Declarations

### Data Availability

The following information was supplied regarding data availability: The raw measurements are available in the Supplemental Files.

## References

[ref-1] Ageberg E, Bennell KL, Hunt MA, Simic M, Roos EM, Creaby MW (2010). Validity and inter-rater reliability of medio-lateral knee motion observed during a single-limb mini squat. BMC Musculoskeletal Disorders.

[ref-2] Binkley JM, Stratford PW, Lott SA, Riddle DL (1999). The Lower Extremity Functional Scale (LEFS): scale development, measurement properties, and clinical application. North American Orthopaedic Rehabilitation Research Network. Physical Therapy.

[ref-3] Boudreau SN, Dwyer MK, Mattacola CG, Lattermann C, Uhl TL, McKeon JM (2009). Hip-muscle activation during the lunge, single-leg squat, and step-up-and-over exercises. Journal of Sport Rehabilitation.

[ref-4] Brydges CR (2019). Effect size guidelines, sample size calculations, and statistical power in gerontology. Innovation in Aging.

[ref-5] Carroll LA, Paulseth S, Martin RL (2022). Forefoot injuries in athletes: integration of the movement system. International Journal of Sports Physical Therapy.

[ref-6] Claiborne TL, Armstrong CW, Gandhi V, Pincivero DM (2006). Relationship between hip and knee strength and knee valgus during a single leg squat. Journal of Applied Biomechanics.

[ref-7] Cornell DJ, Ebersole KT (2018). Intra-rater test-retest reliability and response stability of the fusionetics™ movement efficiency test. International Journal of Sports Physical Therapy.

[ref-8] Crossley KM, Zhang WJ, Schache AG, Bryant A, Cowan SM (2011). Performance on the single-leg squat task indicates hip abductor muscle function. The American Journal of Sports Medicine.

[ref-9] De Vet HC, Terwee CB, Knol DL, Bouter LM (2006). When to use agreement *versus* reliability measures. Journal of Clinical Epidemiology.

[ref-10] Disantis AE, Martin R (2022a). Movement system dysfunction applied to youth and young adult throwing athletes. International Journal of Sports Physical Therapy.

[ref-11] Disantis AE, Martin RL (2022b). Classification based treatment of Greater Trochanteric Pain Syndrome (GTPS) with integration of the movement system. International Journal of Sports Physical Therapy.

[ref-12] Ferrer-Peña R, Calvo-Lobo C, La Touche R, Fernández-Carnero J (2020). Hip-joint posture and movement alterations are associated with high interference of pain in the life of patients with greater trochanteric pain syndrome. Journal of Manipulative and Physiological Therapeutics.

[ref-13] Fridrich J, Brakke R, Akuthota V, Sullivan W (2017). Reliability and practicality of the core score: four dynamic core stability tests performed in a physician office setting. Clinical Journal of Sport Medicine.

[ref-14] Frohm A, Heijne A, Kowalski J, Svensson P, Myklebust G (2012). A nine-test screening battery for athletes: a reliability study. Scandinavian Journal of Medicine & Science in Sports.

[ref-15] Gianola S, Castellini G, Stucovitz E, Nardo A, Banfi G (2017). Single leg squat performance in physically and non-physically active individuals: a cross-sectional study. BMC Musculoskeletal Disorders.

[ref-16] Haley SM, Fragala-Pinkham MA (2006). Interpreting change scores of tests and measures used in physical therapy. Physical Therapy.

[ref-17] Hall MP, Paik RS, Ware AJ, Mohr KJ, Limpisvasti O (2015). Neuromuscular evaluation with single-leg squat test at 6 months after anterior cruciate ligament reconstruction. Orthopaedic Journal of Sports Medicine.

[ref-18] Hansen R, Lundgaard-Nielsen M, Henriksen M (2021). Visual assessment of dynamic knee joint alignment in patients with patellofemoral pain: an agreement study. PeerJ.

[ref-19] Harris-Hayes M, Steger-May K, Koh C, Royer NK, Graci V, Salsich GB (2014). Classification of lower extremity movement patterns based on visual assessment: reliability and correlation with 2-dimensional video analysis. The Journal of Athletic Training.

[ref-20] Hatton AL, Kemp JL, Brauer SG, Clark RA, Crossley KM (2014). Impairment of dynamic single-leg balance performance in individuals with hip chondropathy. Arthritis Care & Research.

[ref-21] Herrington L (2014). Knee valgus angle during single leg squat and landing in patellofemoral pain patients and controls. Knee.

[ref-22] Hollman JH, Galardi CM, Lin IH, Voth BC, Whitmarsh CL (2014). Frontal and transverse plane hip kinematics and gluteus maximus recruitment correlate with frontal plane knee kinematics during single-leg squat tests in women. Clinical Biomechanics.

[ref-23] Junge T, Balsnes S, Runge L, Juul-Kristensen B, Wedderkopp N (2012). Single leg mini squat: an inter-tester reproducibility study of children in the age of 9–10 and 12–14 years presented by various methods of kappa calculation. BMC Musculoskeletal Disorders.

[ref-24] Kaukinen PT, Arokoski JP, Huber EO, Luomajoki HA (2017). Intertester and intratester reliability of a movement control test battery for patients with knee osteoarthritis and controls. Journal of Musculoskeletal and Neuronal Interactions.

[ref-25] Kingston B, Murray A, Norte GE, Glaviano NR (2020). Validity and reliability of 2-dimensional trunk, hip, and knee frontal plane kinematics during single-leg squat, drop jump, and single-leg hop in females with patellofemoral pain. Physical Therapy in Sport.

[ref-26] Kivlan BR, Martin RL (2012). Functional performance testing of the hip in athletes: a systematic review for reliability and validity. International Journal of Sports Physical Therapy.

[ref-27] Koo TK, Li MY (2016). A guideline of selecting and reporting intraclass correlation coefficients for reliability research. Journal of Chiropractic Medicine.

[ref-28] Krause DA, Boyd MS, Hager AN, Smoyer EC, Thompson AT, Hollman JH (2015). Reliability and accuracy of a goniometer mobile device application for video measurement of the functional movement screen deep squat test. International Journal of Sports Physical Therapy.

[ref-29] Lenzlinger-Asprion R, Keller N, Meichtry A, Luomajoki H (2017). Intertester and intratester reliability of movement control tests on the hip for patients with hip osteoarthritis. BMC Musculoskeletal Disorders.

[ref-30] Levinger P, Gilleard WL, Sprogis K (2006). Frontal plane motion of the rearfoot during a one-leg squat in individuals with patellofemoral pain syndrome. Journal of the American Podiatric Medical Association.

[ref-31] Lewis CL, Foch E, Luko MM, Loverro KL, Khuu A (2015). Differences in lower extremity and trunk kinematics between single leg squat and step down tasks. PLOS ONE.

[ref-32] Madhavan S, Shields RK (2011). Neuromuscular responses in individuals with anterior cruciate ligament repair. Clinical Neurophysiology.

[ref-33] Martin RL, Irrgang JJ (2007). A survey of self-reported outcome instruments for the foot and ankle. Journal of Orthopaedic & Sports Physical Therapy.

[ref-34] Martin RL, Takla A, Disantis A, Kohlrieser D, Enseki K, Lifshitz L, Grant L, Bizzini M, Voight M, Ryan M, McGovern R, Tyler T, Steinfeld-Mass Y, Campbell A, Zhang Y (2023). Evaluating functional performance tests in those with non-arthritic intra-articular hip pain: an international consensus statement. International Journal of Sports Physical Therapy.

[ref-35] Mastrigt Nv, Naili JE, Broström EW, Harlaar J, Iversen MD (2017). Inter-rater reliability of movement quality during single limb mini-squat test in adults with knee osteoarthritis. Gait & Posture.

[ref-36] McGovern RP, Christoforetti JJ, Martin RL, Phelps AL, Kivlan BR (2019). Evidence for reliability and validity of functional performance testing in the evaluation of nonarthritic hip pain. The Journal of Athletic Training.

[ref-37] McGovern RP, Martin RL, Christoforetti JJ, Kivlan BR (2018). Evidence-based procedures for performing the single leg squat and step-down tests in evaluation of non-arthritic hip pain: a literature review. International Journal of Sports Physical Therapy.

[ref-38] McGovern RP, Martin RL, Phelps AL, Kivlan BR, Nickel B, Christoforetti JJ (2020). Conservative management acutely improves functional movement and clinical outcomes in patients with pre-arthritic hip pain. The Journal of Hip Preservation Surgery.

[ref-39] Mehta SP, Fulton A, Quach C, Thistle M, Toledo C, Evans NA (2016). Measurement properties of the lower extremity functional scale: a systematic review. Journal of Orthopaedic & Sports Physical Therapy.

[ref-40] Naal FD, Impellizzeri FM, Leunig M (2009). Which is the best activity rating scale for patients undergoing total joint arthroplasty?. Clinical Orthopaedics and Related Research.

[ref-41] Nae J, Creaby MW, Cronstrom A, Ageberg E (2017). Measurement properties of visual rating of postural orientation errors of the lower extremity—a systematic review and meta-analysis. Physical Therapy in Sport.

[ref-42] Nakagawa TH, Moriya ET, Maciel CD, Serrao FV (2012). Trunk, pelvis, hip, and knee kinematics, hip strength, and gluteal muscle activation during a single-leg squat in males and females with and without patellofemoral pain syndrome. Journal of Orthopaedic & Sports Physical Therapy.

[ref-43] Ortqvist M, Mostrom EB, Roos EM, Lundell P, Janarv PM, Werner S, Brostrom EW (2011). Reliability and reference values of two clinical measurements of dynamic and static knee position in healthy children. Knee Surgery, Sports Traumatology, Arthroscopy.

[ref-44] Poulsen DR, James CR (2011). Concurrent validity and reliability of clinical evaluation of the single leg squat. Physiotherapy: Theory and Practice.

[ref-45] Ressman J, Grooten WJA, Rasmussen Barr E (2019). Visual assessment of movement quality in the single leg squat test: a review and meta-analysis of inter-rater and intrarater reliability. BMJ Open Sport & Exercise Medicine.

[ref-46] Rolfson O, Eresian Chenok K, Bohm E, Lübbeke A, Denissen G, Dunn J, Lyman S, Franklin P, Dunbar M, Overgaard S, Garellick G, Dawson J (2016). Patient-reported outcome measures in arthroplasty registries. Acta Orthopaedica.

[ref-47] Shrout PE, Fleiss JL (1979). Intraclass correlations: uses in assessing rater reliability. Psychological Bulletin.

[ref-48] Stickler L, Finley M, Gulgin H (2015). Relationship between hip and core strength and frontal plane alignment during a single leg squat. Physical Therapy in Sport.

[ref-49] Stratford PW, Riddle DL (2012). When minimal detectable change exceeds a diagnostic test-based threshold change value for an outcome measure: resolving the conflict. Physical Therapy.

[ref-50] Terwee CB, Bouwmeester W, Van Elsl SL, De Vet HC, Dekker J (2011). Instruments to assess physical activity in patients with osteoarthritis of the hip or knee: a systematic review of measurement properties. Osteoarthritis Cartilage.

[ref-51] Weeks BK, Carty CP, Horan SA (2012). Kinematic predictors of single-leg squat performance: a comparison of experienced physiotherapists and student physiotherapists. BMC Musculoskeletal Disorders.

[ref-52] Whatman C, Hing W, Hume P (2012). Physiotherapist agreement when visually rating movement quality during lower extremity functional screening tests. Physical Therapy in Sport.

[ref-53] Whatman C, Hume P, Hing W (2013). The reliability and validity of physiotherapist visual rating of dynamic pelvis and knee alignment in young athletes. Physical Therapy in Sport.

[ref-54] Willson JD, Davis IS (2008). Lower extremity mechanics of females with and without patellofemoral pain across activities with progressively greater task demands. Clinical Biomechanics.

[ref-55] Zarzycki R, Malloy P, Eckenrode BJ, Fagan J, Malloy M, Mangione KK (2022). Application of the 4-element movement system model to sports physical therapy practice and education. International Journal of Sports Physical Therapy.

[ref-56] Zhang Y, Ai D, Gao Y, Li W, Yi Y, Xu X, Hu H, Zhang N, Yang S, Lian X, Wang Y, Martin Pt RL, Zhang X (2025a). Test-retest reliability and responsiveness for the simplified chinese lower extremity functional scale in patients with lower extremity musculoskeletal injuries. Orthopaedic Journal of Sports Medicine.

[ref-57] Zhang Y, Gao Y, Ai D, Li W, Zhang X, Xu X, Zhang N, Yi Y, Hu H, Yang S, Martin RL, Lian X (2025b). Defining region and duration-specific substantial clinical benefit values for the simplified Chinese lower extremity functional scale. BMC Musculoskeletal Disorders.

[ref-58] Zhang Y, Liu Y, Pan Z, Gao H, Martin RL, Huang X (2025c). Comparing the reliability of the single leg squat test using two, three, and four category ordinal rating scales. PeerJ.

[ref-59] Zhang Y, Zang Y, Martin RL (2025). Clinically most relevant psychometric properties of the lower extremity functional scale: a systematic review. Disability Rehabilitation.

