# Supplementary material for: The reliability and validity of a visual analog scale for rating the single leg squat test
Source: PeerJ. 2026 Jul 28;14:e21600. doi: 10.7717/peerj.21600 (PMC13426336; doi:10.7717/peerj.21600)
Supplement: Appendix S1 [file peerj-14-21600-s001.docx]

Appendix

Description of the Single-Leg Squat Test (SLST) Procedure


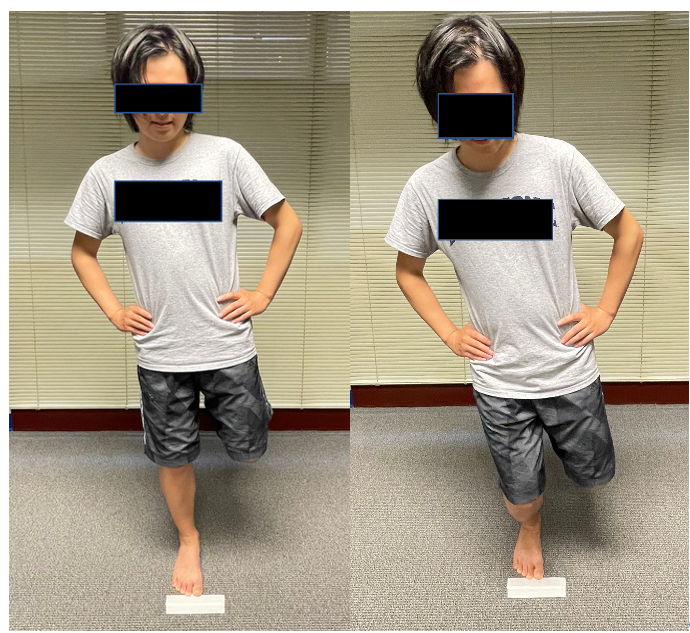


The calculation of angles during 2-D video analysis

Lateral Trunk Motion

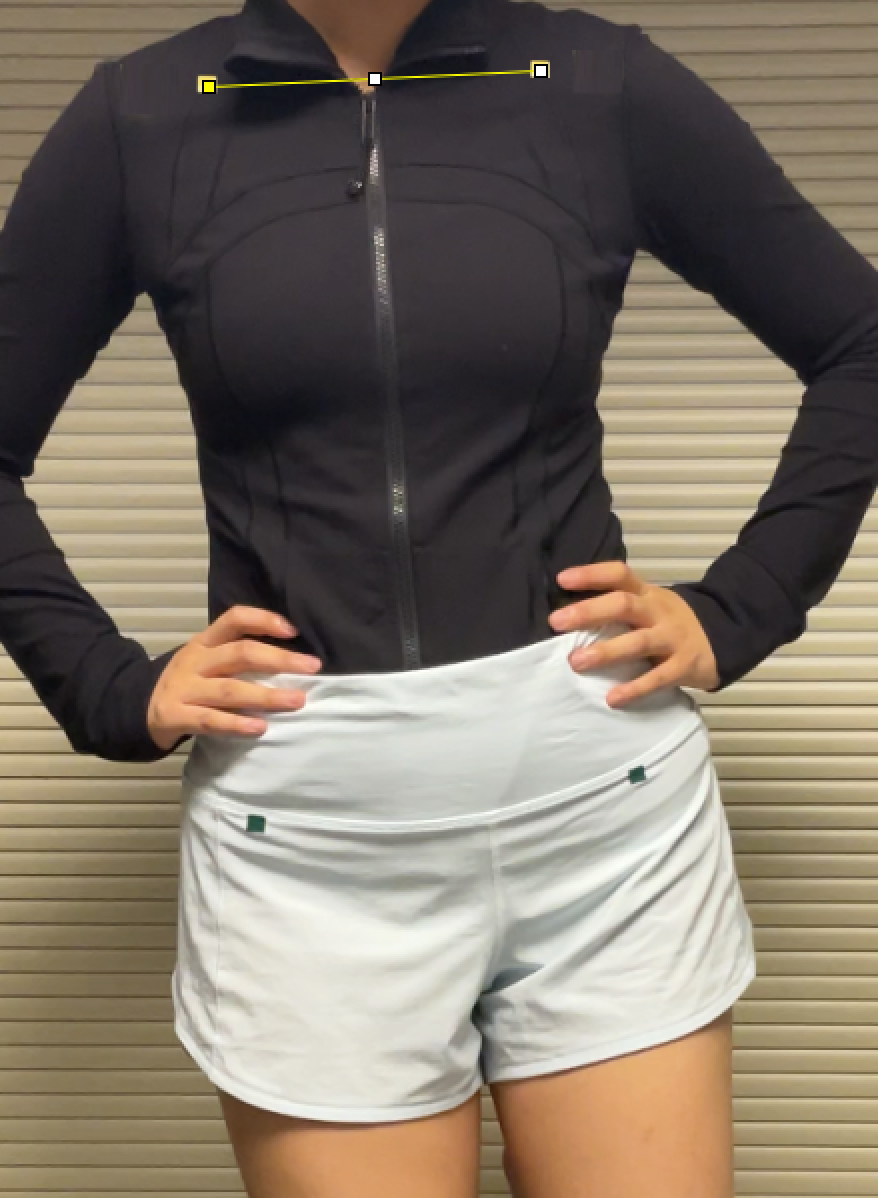

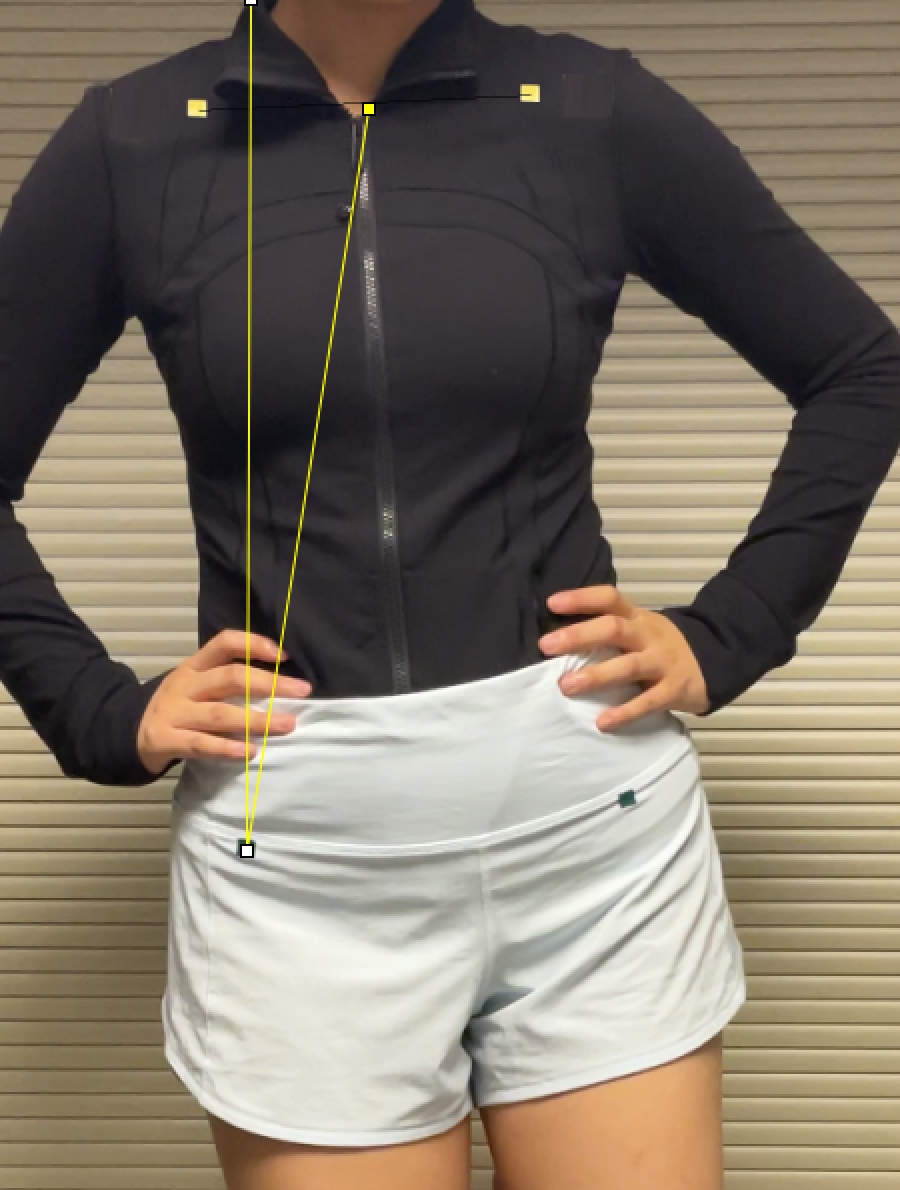


Hip Frontal Plane Projection Angle

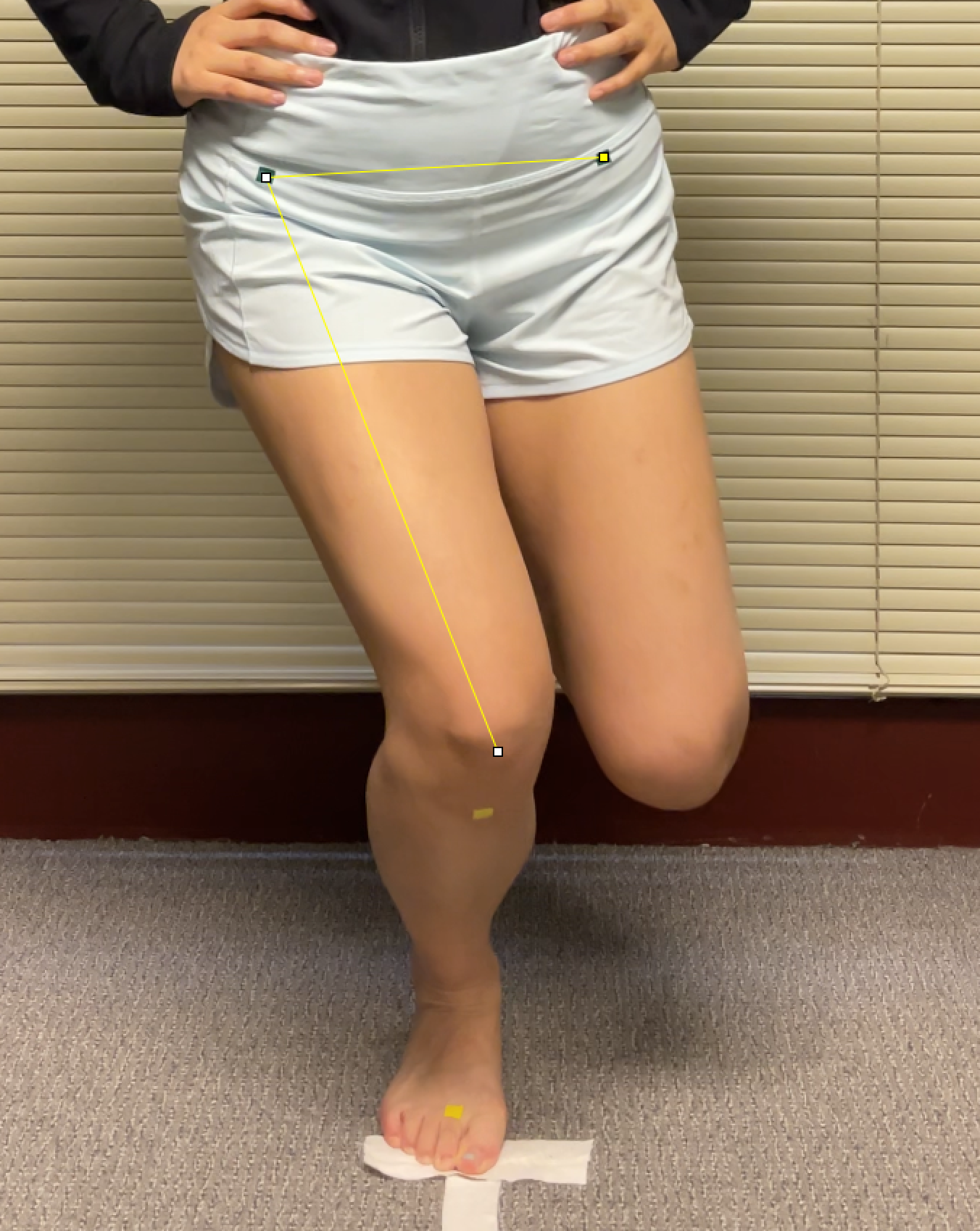

Knee Frontal Plane Projection Angle

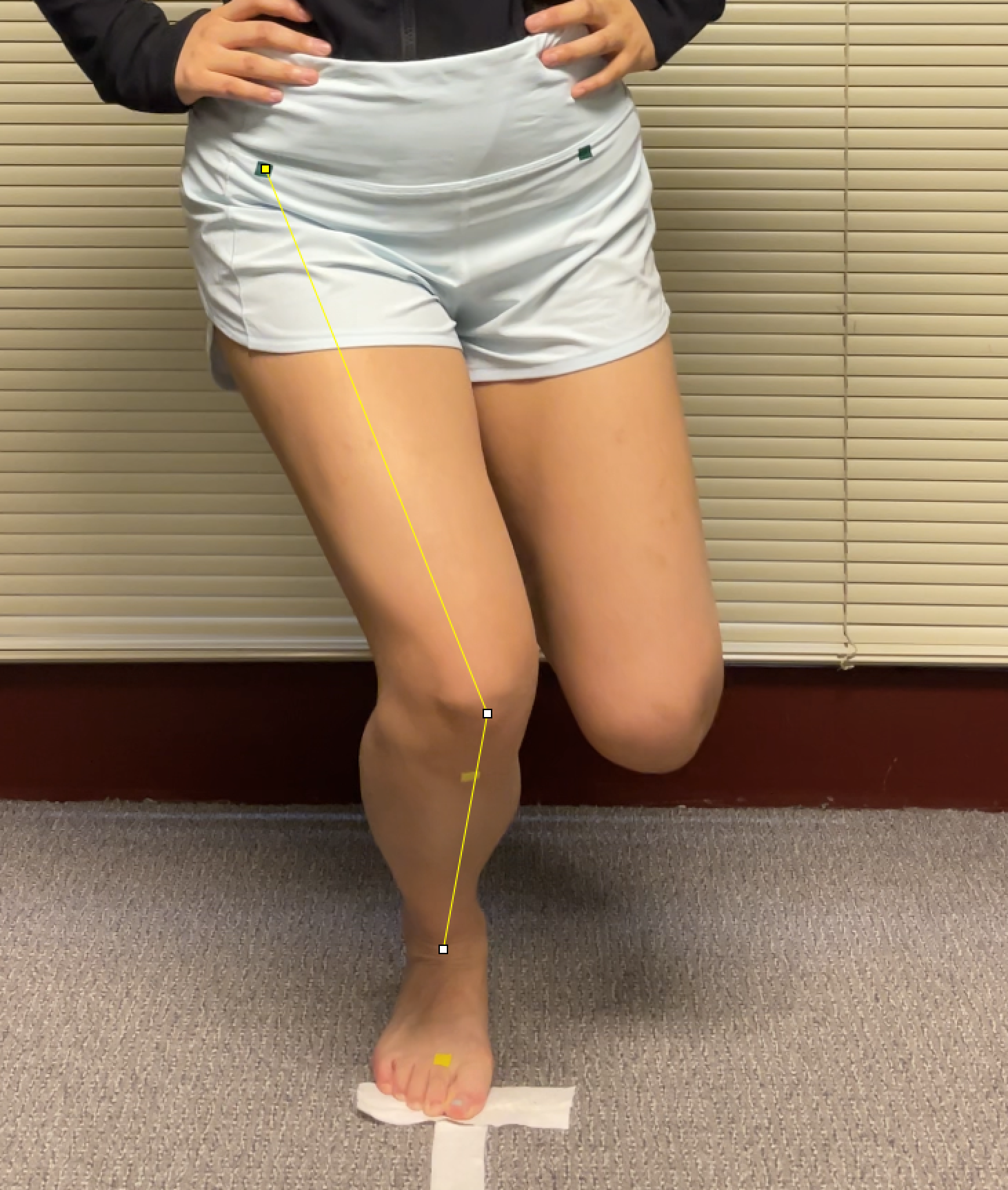


Correlation between Visual Analog Scale Scores and 2-D Kinematic Data

The inter-rater reliability between the two researchers who independently extracted kinematic data from the 2-D video

| Components | ICC (95%CI) | | SEM (degrees) |
| --- | --- | --- | --- |
| Trunk lateral flexion | | 0.97 (0.95, 0.98) | 0.6 |
| Hip adduction | | 0.95 (0.9, 0.97) | 1.7 |
| Dynamic knee valgus | | 0.97 (0.87,0.98) | 0.7 |

CI: confidence interval SEM: Standard error of measurement
